# Windowed persistent homology: A topological signal processing algorithm applied to clinical obesity data

**DOI:** 10.1371/journal.pone.0177696

**Published:** 2017-05-12

**Authors:** Craig Biwer, Amy Rothberg, Heidi IglayReger, Harm Derksen, Charles F. Burant, Kayvan Najarian

**Affiliations:** 1 Department of Computational Medicine and Bioinformatics, University of Michigan, Ann Arbor, MI, United States of America; 2 Department of Internal Medicine, University of Michigan, Ann Arbor, MI, United States of America; 3 Department of Mathematics, University of Michigan, Ann Arbor, MI, United States of America; Montclair State University, UNITED STATES

## Abstract

Overweight and obesity are highly prevalent in the population of the United States, affecting roughly 2/3 of Americans. These diseases, along with their associated conditions, are a major burden on the healthcare industry in terms of both dollars spent and effort expended. Volitional weight loss is attempted by many, but weight regain is common. The ability to predict which patients will lose weight and successfully maintain the loss versus those prone to regain weight would help ease this burden by allowing clinicians the ability to skip treatments likely to be ineffective. In this paper we introduce a new windowed approach to the persistent homology signal processing algorithm that, when paired with a modified, semimetric version of the Hausdorff distance, can differentiate the two groups where other commonly used methods fail. The novel approach is tested on accelerometer data gathered from an ongoing study at the University of Michigan. While most standard approaches to signal processing show no difference between the two groups, windowed persistent homology and the modified Hausdorff semimetric show a clear separation. This has significant implications for clinical decision making and patient care.

## Introduction

As medical monitoring devices continue to grow in complexity and shrink in size, both the number of possible concurrent measurements and the size of the observable population increase. These factors in turn result in a rise in the amount of data available for analysis, which is driving the need for new processing algorithms. The sheer volume of recorded values makes it difficult to process within a relevant timeline using conventional means, and the intricacies of some of the more obscure variables makes them difficult to interpret at all. Because of this, the need for novel signal processing algorithms that can detect and highlight underlying subtleties and features is becoming ever more apparent. Weight management is one area in which new methods could be applied to great effect. Overweight and obesity among Americans are highly prevalent, affecting roughly 2/3 of the country’s population [1]. Obesity is a major risk factor for cardiometabolic diseases such as type 2 diabetes, hypertension, obstructive sleep apnea, and a variety of other disabling conditions, all of which combine to an impressive burden on the healthcare industry not only in terms of dollars spent but also effort and time expended. Volitional weight loss is attempted by the majority of obese individuals, but a variety of neurobehavioral mechanisms are activated following weight loss that result in weight regain in most instances [2]. However, in most controlled weight loss studies, a proportion of individuals can maintain a significant weight loss [3, 4]. To date, no definitive factors have been identified that can predict weight loss or weight regain. With the emerging monitoring capabilities, though, datasets large enough to investigate are becoming possible. The ability to differentiate between patients who will maintain weight loss and those prone to weight regain would not only allow for a greater understanding of the biology of obesity but also provide a path to tailored interventions in the population. Early interventions in those at high risk have been shown to reduce the risk of diseases such as those mentioned above [5].

This problem, along with many others in the clinical setting where time-series measurements are to be analyzed, stands to benefit from advanced signal processing. A common practice in the medical field, various signal processing algorithms have been applied to a wide variety of situations. From heart rate monitoring to myoelectric signal classification, various techniques are used to analyze time series data. Feature extraction has proved to be an effective approach, commonly used in health applications, and involves calculating variables characteristic of the signal. Another approach, direct comparison methods, analyzes and compares raw signals simultaneously. The power, entropy, and average value of a signal are three common features used in signal processing, and the Pearson correlation coefficient is used when comparing signals and samples directly. The currently used methods, while effective in many applications, cannot always differentiate between two sets of similar signals in complex health applications, especially when the differences among signals are subtle. Indeed, the main shortcoming of existing models in predicting weight maintenance is a clear lack of effective computational approaches to mining available data. Instead, most of the existing predictive models of obesity are based on correlation of weight regain/loss with only a small amount of basic patient information, which result in models with limited predictive capabilities. For instance, a major factor that can help lead the personalization of treatments for obesity is estimation of the type and level of physical activities. However, the motion signals, when analyzed with the conventional signal processing methods, have failed to produce accurate and robust prediction results. The complexity of the data collected for any one patient, let alone that found in the collective data of a large study groups, demands more advanced computational techniques that can extract these subtle patterns and distinguish sub-classes of weight loss and regain. In this study we describe a new approach in signal processing that can detect subtle changes in the behavior of complex signals, in particular motion time-series, and as such distinguish between patient cohorts with different clinical outcome. The proposed approach is based on our extended formulation of the persistent homology theory and introduction of a modified, semimetric version of the Hausdorff distance to analyze data in the feature space [6–12]. We apply and demonstrate the efficacy of the proposed computational methods in differentiating between various levels of weight loss maintenance.

## Materials and methods

### Data

This study was reviewed and approved by the University of Michigan Institutional Review Board and all study participants provided written, informed consent. Additionally, after data collection, all data files were de-identified and curated prior to analysis. In this study, we exploit the unique infrastructure and database of the University of Michigan Weight Management Program clinic (MWMP), a demonstration unit of the NIH-funded Michigan Nutrition Obesity Research Center. This study was reviewed and approved by the Institutional Review Board of the University of Michigan (HUM00030088). The MWMP is a structured two-year, multicomponent, multidisciplinary lifestyle intervention which employs intensive caloric restriction for the first 12 weeks to promote 15% weight loss, followed by interventions and routine follow-ups to support long-term behavior change in diet and physical activity [13]. Participants completed a battery of clinical, psychological and metabolic assessments before (baseline)—Phase 1, after intensive weight loss (3–6 months)—Phase 2, and at the end of two years—Phase 3. In addition, participants were routinely asked to wear a tri-axial accelerometer that also measures galvanic skin response and near body ambient temperature (BodyMedia SenseWear armbands, http://www.bodymedia.com) for a period of 7 days, only removing the monitor to charge it while participating in water activities (e.g. showering, swimming). Each test yielded an individual file, which resulted in a large number of disparate data sources for each participant. Each individual, before testing began, gave his or her informed consent that the collected data be used for research purposes. In addition, after collection, all data files were de-identified and curated prior to analysis. This pre-processing also involved automatic and manual error-checking. Once all the relevant patient data were parsed, the files were spot-checked for inconsistencies. This was done by plotting various data values from numerous participant files and checking for outliers.

While worn, the armbands recorded the number of peaks in the accelerometer signal, once per minute, for each of three dimensions: transverse, forward, and longitudinal. These three numbers were summed each minute, resulting in a roughly week-long general movement profile for each individual. In this study, only participants who wore the device for at least 7000 minutes are included. This meant each individual wore the device for at least 16.5 hours per day for the full week, or approximately 23 hours per day for five days. This number was chosen as it yielded as long a signal as possible while including as many participants as possible. Any participant for whom 7000 minutes of data were not recorded was excluded from this analysis, as well as those that had not yet progressed far enough into the study to have measurable results. Each included signal was cropped at the 7000 minute mark, resulting in a uniform length across all studied movement profiles. This was done for a number of reasons: the set length allowed for uniform mapping of the data without the need to stretch or skew the signals, preventing any interference from different time resolutions; no signal included more potential information than another, as they were all of equal length. A final inclusion requirement was that the participant be classified as a ‘success’ or ‘failure’. As the specific aim of this study was to predict weight-loss maintenance, each participant was given a label based on weight loss and regain. If an individual failed to lose at least 15% of his or her starting body weight between Phase 1 and Phase 2, that person was labeled a ‘failure’. Those who succeeded in achieving that goal, and who finished Phase 3 at a weight no greater than 90% of his or her starting weight, were labeled a ‘success’. Any individual who completed the study without maintaining at least a 10% weight loss was considered a ‘failure’. As a result, the individuals included in the study had all successfully completed at least Phases 1 and 2, and some had finished Phase 3. Accounting for all of the above criteria, 100 participants were included in this study. This cohort consisted of 36 males and 64 females with an average age of 50 ± 9 years old.

Next, our computational analysis based on persistent homology is described. A windowed formulation of persistent homology was used to extract characteristic features from the data from each participant. These features, represented as persistence diagrams, were then used to predict success. The ability of persistent homology-based features to predict success/failure was statistically analyzed, as described later.

### Signal analysis using persistent homology

Persistent homology is a broad mathematical theory, and one of its applications is examining how the characteristics of an object in a space change based on the spatial resolution used to examine the object. As the resolution changes, persistent homology features, representing the special characteristics of the object, quantify these changes. The transitions in these features can be studied to help develop a better understanding of the object. When applied to time series as objects, persistent homology can be used to extract features of a signal representing the changes in the characteristic patterns of variations observed in the signal at different resolutions [14–21]. Specifically, the persistent homology algorithm converts a signal into points scattered across a min-max plane. By treating each minimum in a time-series as the ‘birth’ of a feature and each maximum as a ‘death’ it is possible to examine the significance of a trend by the persistence of its corresponding min-max pairing. Larger differences between two extrema correspond to more pronounced variation, and any resulting points will be farther from the *y* = *x* diagonal. Conversely, a point closer to the diagonal represents a smaller magnitude of change and is more likely to be noise. The resulting min-max plot, or ‘persistence diagram’, represents the characteristics of the input sequence and can be used to compare the differences between the patterns and variations of signals. This information can also be visualized in a ‘barcode’ format, as described in [22]. The process, including the derivation of the persistence diagram, is as follows:

Suppose that *f* is a real-valued function on the discrete set {1,2,…,*n*}. To make notation convenient, we also define *f*(0) = ∞ and *f*(*n*+1) = −∞. Also, we modify *f* to define a function $\tilde{f}$ defined by $\tilde{f}\left(i\right)=f\left(i\right)+\varepsilon i$ where *ε* > 0 is infinitesimal. We define the function-value ordering ⊑ on {0,1,…,*n*+1} as follows. If 0 ≤ *i*, *j* ≤ *n*+1 then we define *i* ⊑ *j* if $\tilde{f}\left(i\right)\leq \tilde{f}\left(j\right)$. Equivalently, we have *i* ⊑ *j* if *f*(*i*)<*f*(*j*), or *f*(*i*) = *f*(*j*) and *i* < *j*. The relation ⊑ on {0,1,…,*n*+1} is a total ordering. So for all *i* and *j* we have:

1. *i* ⊑ *i*;
2. *i* ⊑ *j* or *j* ⊑ *i*;
3. if *i* ⊑ *j* and *j* ⊑ *i*, then *i* = *j*;
4. if *i* ⊑ *j* and *j* ⊑ *k* then *i* ⊑ *k*.

We also write *i* ⊏ *j* if *i* ⊑ *j* and *i* ≠ *j*. Define the set of local minima and maxima by

$$\begin{array}{c} E_{\text{min}}=\{i|1\leq i\leq n,\;i⊏i-1,i⊏i+1\} \end{array}$$ (1a)

$$\begin{array}{c} E_{\text{max}}=\{i|1\leq i\leq n,\;i-1⊏i,\;i+1⊏i\} \end{array}$$ (1b)

respectively.

**Lemma 1**. *The sets E*_min_ *and* *E*_max_ *have the same number of elements*.

*proof*. It is not hard so see that the smallest element of the set of extrema *E* = *E*_min_ ∪ *E*_max_ with respect to the ordering ≤ lies in *E*_min_. Also, the largest element lies in *E*_max_. It is also elementary to see that local maxima and local minima alternate. So the number of local maxima and local minima is the same.

By the lemma, there exists *r*, *a*_1_, *a*_2_,…,*a*_*r*_, *b*_1_, *b*_2_,…,*b*_*r*_ such that *E*_min_ = {*a*_1_, *a*_2_,…,*a*_r_} and *E*_max_ = {*b*_1_, *b*_2_,…,*b*_r_} such that *a*_1_ ⊏ *a*_2_ ⊏ ⋯ ⊏ *a*_*r*_ and *b*_1_ ⊏ *b*_2_ ⊏ ⋯ ⊏ *b*_*r*_. The proof of the lemma shows that there are permutations *τ* and *γ* in the symmetric group *S*_*r*_ such that

$$\begin{array}{c} a_{\tau (1)}<b_{\gamma (1)}<a_{\tau (2)}<b_{\gamma (2)}<\cdots <a_{\tau (r)}<b_{\gamma (r)}. \end{array}$$

**Lemma 2**. *We have a*_*i*_ ⊏ *b*_*i*_ *for all i*.

*proof*. It is clear that *a*_*τ*(*i*)_ ⊏ *b*_*γ*(*i*)_ for all *i*. For *j* ≤ *i* we have *a*_*γ*^−1^*τ*(*j*)_ ⊏ *b*_*j*_ ⊑ *b*_*i*_. So at least *i* of the *a*’s are smaller than *b*_*i*_ with respect to ⊏. This implies that *a*_*i*_ ⊏ *b*_*i*_.

If a,b∈R then we define

$$\begin{array}{c} \left(a,b\right)=\left\{\begin{array}{cc} \{x\in R|a<x<b\}, & \text{if}\;a\leq b\\ \{x\in R|b<x<a\}, & \text{if}\;a>b \end{array}\right. \end{array}$$ (2)

We define a permutation *σ* ∈ *S*_*r*_ and sets *U*_1_,*U*_2_,…,*U*_*r*_ inductively as follows:

$$\begin{array}{c} U_{k}=\left\{i\;|\;1\leq i\leq r,\;a_{i}⊏b_{k},\;i\notin \left\{\sigma \left(1\right),\sigma \left(2\right),\ldots ,\sigma \left(k-1\right)\right\}\right\} \end{array}$$ (3)

and

$$\begin{array}{c} \sigma \left(k\right)=\max\left\{i|i\in U_{k}\;\text{and}\;\text{for}\;\text{all}\;j\in U_{k}\;\text{with}\;j<i\;\text{we}\;\text{have}\;a_{j}\notin \left(a_{i},b_{k}\right)\right\}. \end{array}$$ (4)

By Lemma 2, the set *U*_*k*_ is nonempty.

**Definition 1**. The persistence diagram associated to the function *f* is

$$\begin{array}{c} \{\left(f\left(a_{\sigma (1)}\right),f\left(b_{1}\right)\right),\left(f\left(a_{\sigma (2)}\right),f\left(b_{2}\right)\right),\ldots ,\left(f\left(a_{\sigma (r)}\right),f\left(b_{r}\right)\right)\}. \end{array}$$

**Fig 1:**
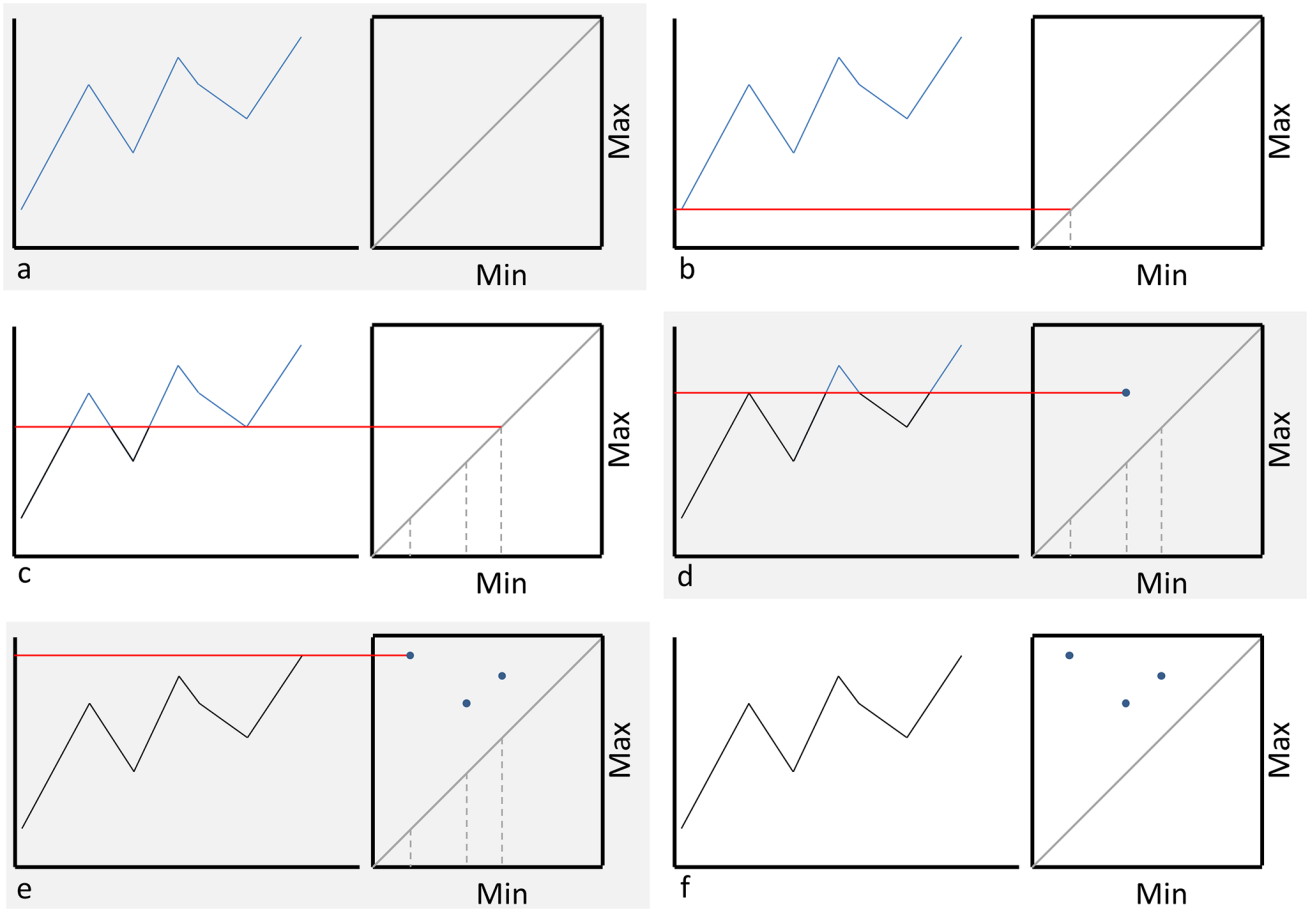
Persistent homology.

The mathematical complexity of the persistent homology approach formulated above might mask its great conceptual potentials to analyze complex signals. Below is a graphical illustration and explanation of this method. As shown in Fig 1, first a given signal is plotted and all local extrema (maxima and minima) are identified. A square plot in the min-max plane is generated and placed such that the vertical axes align between the two graphs, and the line *y* = *x* is drawn (Fig 1a). Starting with the smallest minimum (in this case, point 1), a mark is placed on the min axis of the second graph corresponding to the minimum’s y value (Fig 1b). Each local extremum is considered, moving from smallest to largest, and for each minimum another mark is placed (Fig 1c). When a maximum is encountered, that point’s y value is paired with the mark from the most recent minimum, as long as there is no other maximum between the two points. If such a maximum exists, the second most recent minimum is considered, and the process continues until a suitable pairing is found (Fig 1d). The next extremum is then considered, and the algorithm proceeds until all points are paired (Fig 1e). In the event that there are more minima than maxima (or vice versa), the point with the largest x value in the larger set is dropped. Once completed, the points in the min-max plane constitute the signal’s persistence diagram (Fig 1f). As it can be seen, starting from a time-series, persistent homology creates a pattern of dots in the max-min plot that represent the variations of the signal.

These min-max patterns represent how the variations in signals can be used, as described later, to effectively distinguish between different types of signals (e.g. signals representing ‘success’ and those representing ‘failure’). Next, we describe our method of distinguishing between different patterns/signals by using a measure that quantifies the disparity between different min-max plots.

### Assessment of feature space using modified Hausdorff semimetric and Wasserstein distance

The persistent homology method can be used either as a stand-alone analysis tool or as a comparative metric. For the former case, the persistence diagram can be examined and features extracted, and for the latter two persistence diagrams can be compared in a number of different ways. For this study, persistent homology was used to compare different armband activity signals. However, calculating and comparing the persistence diagrams for the full 7000 points from each input is not only computationally expensive but also masks some important patterns in parts of the signals due to the averaging effect. Moreover, for such long signals, not only would the persistence diagrams be extremely dense but any order to the signals would be lost: points generated by pairings of extrema from the beginning of one signal could be near points generated by extrema at the end of the second signal. In addition, the dots themselves may result in inaccurate interpretation or analysis of the signal. For instance, pairing a minimum from near the beginning of the signal with a maximum from near the end would be relating two otherwise independent measurements: the activity from the first point would be entirely separate from the activity that generated the second. As such, comparing the persistence diagrams of two long signals would yield little valuable information. To solve this problem the armband signals were first broken into windows and the algorithm was applied to each window. This had the added benefit of decreasing the computational time of our implementation by a factor of over 300.

In this study we designed and implemented a windowed based approach to persistent homology to address the above mentioned issues. Specifically, each 7000-point signal was broken into 350 windows, each containing 20 points. This was done by simply splitting the original signal into equally-sized standalone segments using a rectangular window: no overlap or tapering was used. The window size was chosen because it allowed for reasonable variation within a window while at the same time ensuring that any two paired points would be closely related in time. A persistence diagram was calculated for each window, and corresponding windows from two signals were compared (i.e. the first window from each signal was paired, followed by the second, etc.).

Various metrics exist for calculating the distance between two sets of points, including the Hausdorff distance and the q-th Wasserstein distance. The latter is defined in [23] between two sets *A* and *B* as:

$$\begin{array}{c} W_{q}\left(A,B\right)={\left[\underset{f:A\to B}{\inf}\sum_{a\in A}||a-{f\left(b\right)||}_{\infty }^{q}\right]}^{1/q} \end{array}$$ (5)

where *f* is the set of all bijections *X* → *Y*. When *q* = 1, this metric reduces to the minimum possible sum of the distances between each point in *X* and its corresponding point in *Y*. By allowing points to map to the *y* = *x* line, any contribution from noise is minimal. The Hausdorff distance *d*_*H*_ is calculated as:

$$\begin{array}{c} d_{H}\left(A,B\right)=\text{max}\left\{\underset{a\in A}{\sup}\min_{b\in B}d\left(a,b\right),\underset{b\in B}{\sup}\min_{a\in A}d\left(a,b\right)\right\} \end{array}$$ (6)

where *d*(*a*, *b*) is the Euclidean distance between points *a* and *b*. However, this method is quite sensitive to outliers: one anomalous point in either set could greatly skew the measured value. Due to the stochastic and noisy nature of the data being examined, the metric used to compare two persistence diagrams had to be tolerant of such deviations and outliers; if it was not, one large peak caused by on outlier could alter the distance. This led us to the use of a modified, semimetric version of the Hausdorff distance [12]. Replacing the inner suprema with an average gives:

$$\begin{array}{c} d_{mH}\left(A,B\right)=\text{max}\left\{\frac{1}{\left|A\right|}\sum_{a\in A}\min_{b\in B}d\left(a,b\right),\frac{1}{\left|B\right|}\sum_{b\in B}\min_{a\in A}d\left(a,b\right)\right\} \end{array}$$ (7)

This metric is much more tolerant of outliers as they are included as one part in a general sum and not the only representative number. However, as noted in [12], this version is not a true distance metric as it does not satisfy the triangle inequality. As such, because it satisfies the other distance metric requirements, this comparison qualifies as a semimetric.

## Results

To begin, the power of each raw armband data was calculated (Table 1). This rather simple and intuitive feature is often used in analysis of activity signals as a main characteristic number describing the data. In this study, however, there is no measurable difference between the power of a participant labeled as a failure and that of a participant labeled as a success (*p* = 0.326). The sample size was 100, of which 79 were labeled ‘failures’ and the remaining 21 were considered ‘successes’.

**Table 1:** 

	Label	Average	Standard Deviation
**Power** (*p* = 0.326)	Failures	196205	56942
	Successes	182888	46034
**Entropy** (*p* = 0.608)	Failures	0.6560	0.1433
	Successes	0.6732	0.1037
**Average** (*p* = 0.262)	Failures	289.9	57.1
	Successes	274.3	53.0

The entropy and average of signals, also popular in assessment of activity signals, were calculated and analyzed as well (Table 1). Entropy as a measure of disorder and information can often distinguish between functional classes of data, in particular when dealing with biomedical signals, and is heavily used in the signal processing literature. However, as in the case of power, there was no significant difference between the failure and success groups (*p* = 0.608 and *p* = 0.262, respectively).

As a final check using current established methods, correlation coefficient calculation was used to analyze the data. For this, each signal was compared to every other signal in the pool and a correlation value was obtained. When armband data from two individuals labeled as failures were compared, the resulting signal was placed into a ‘failure vs failure’ group (N = 3081); likewise, when the movement profiles of two successes were compared, the result was placed into a ‘success vs success’ group (N = 210). When comparing two ‘failure’ signals, the average correlation coefficient was 0.0712, while the average for comparing two ‘success’ signals was 0.0992. While the standard deviations were relatively high, as shown in Table 2, there was a statistically relevant difference between the groups (*p* = 0.006).

**Table 2:** 

Correlation (*p* = 0.006)	Average	Standard Deviation
Failure vs Failure	0.0712	0.1431
Failure vs Success	0.0864	0.1376
Success vs Success	0.0992	0.1158

Comparing the ‘failure vs success’ group (N = 1659) to the ‘failure vs failure’ group yields another statistically significant difference (*p* = 0.0004), but comparing it to the ‘success vs success’ group does not (*p* = 0.1968). This indicates that the failures share less intra-group similarities than do the successes, but the second two cases are indistinguishable under this metric.

**Fig 2:**
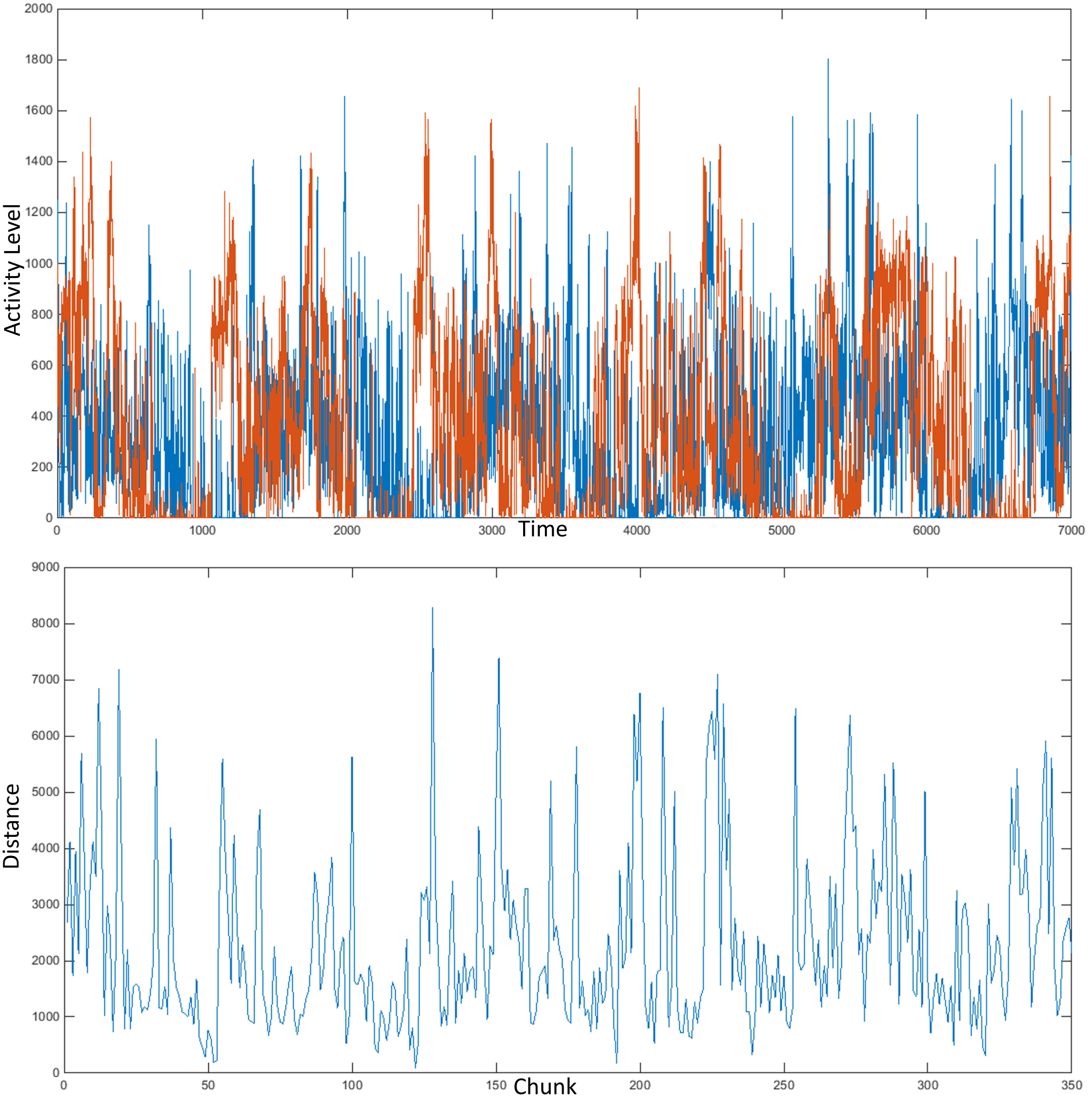
Data analysis.

Next, the armband data of each of the 100 participants was compared to every other file using our proposed windowed persistent homology method. Persistence diagrams were generated for each of the 350 windows extracted from a signal. These plots were compared to corresponding plots from a second signal using the modified Hausdorff semimetric, and a single number was noted for each window. The result of the algorithm, when applied to two armband/activity files, was a new signal with a length of 350 points, corresponding to the distance between the two input plots over time. The top of Fig 2 shows two armband signals plotted together, one drawn in red and the other in blue. Plotting the measured distance between two corresponding windows over the course of the analysis yields the signal shown in the bottom of Fig 2.

The average value of the resulting signal was placed into a group based on the input signals, again separating ‘failure vs failure’, ‘failure vs success’, and ‘success vs success’. The average of these averages was then calculated and this was used as the characteristic value for each group (Table 3). Using an unpaired t test, the analysis showed that there is a statistically significant difference between not only the two main groups (*p* = 1.505^−23^) but between any two of the three (*p* = 1.661 × 10^−28^ and *p* = 5.715^−9^ for ‘failure vs failure’ vs ‘failure vs success’ and ‘failure vs success’ vs ‘success vs success’, respectively). It should be noted that the smaller average distance between successes when compared to that between failures is further strengthened by the correlation analysis: while the coefficients were small, the successes tended to be more highly correlated with one another than did the failures.

**Table 3:** 

Per. Hom. (*p* = 1.505 × 10^−23^)	Average	Standard Deviation
Failure vs Failure	378.27	64.79
Failure vs Success	356.99	58.59
Success vs Success	332.58	41.29

These results were consistent across variations in window size: values from 15 to 25 were also tried, with p-values no worse than an order of magnitude higher (*p* = 1.170 × 10^−8^ for ‘failure vs success’ vs ‘success vs success’ with a window size of 16); some comparisons were more significant. Additionally, the method was implemented on ‘blind’ files in which the labels were randomly generated. With this random distribution there were no statistically significant differences between the groups (average *p* = 0.113 over 100 trials for the main pairing), further reinforcing the notion that our method is capturing some difference in the underlying structures of the signals.

Finally, we applied the same approach but this time used the q-th Wasserstein true distance metric to compare the persistence diagrams (q equal to one; code provided by [23] was used). As show in Table 4, the results are again statistically significant when comparing the ‘failure vs failure’ group to the ‘success vs success’ group (*p* = 1.231 × 10^−5^). While comparing the ‘failure vs failure’ cohort to the ‘failure vs success’ set also yields a significant difference (*p* = 6.181 × 10^−7^), it should be noted that both p-values are larger than their counterparts obtained using the semimetric. In addition, this true metric does not detect a measurable variation between the ‘failure vs success’ and ‘success vs success’ groups (*p* = 0.012). When run with randomly generated labels, the results are once again not significant for any combination of groups (average *p* = 0.084 over 100 trials for the main pairing).

**Table 4:** 

q-Wass. (*p* = 1.241 × 10^−5^)	Average	Standard Deviation
Failure vs Failure	711.88	63.17
Failure vs Success	702.63	56.36
Success vs Success	692.48	44.47

Despite the lack of complete statistical significance between all pairs, the pattern of higher intra-group similarities in the successes than the failures is continued. This, combined with the results of the ‘blind’ files, lends even more credence to the claim that there is an underlying disparity between the behaviors of the two groups.

## Discussion

**Fig 3:**
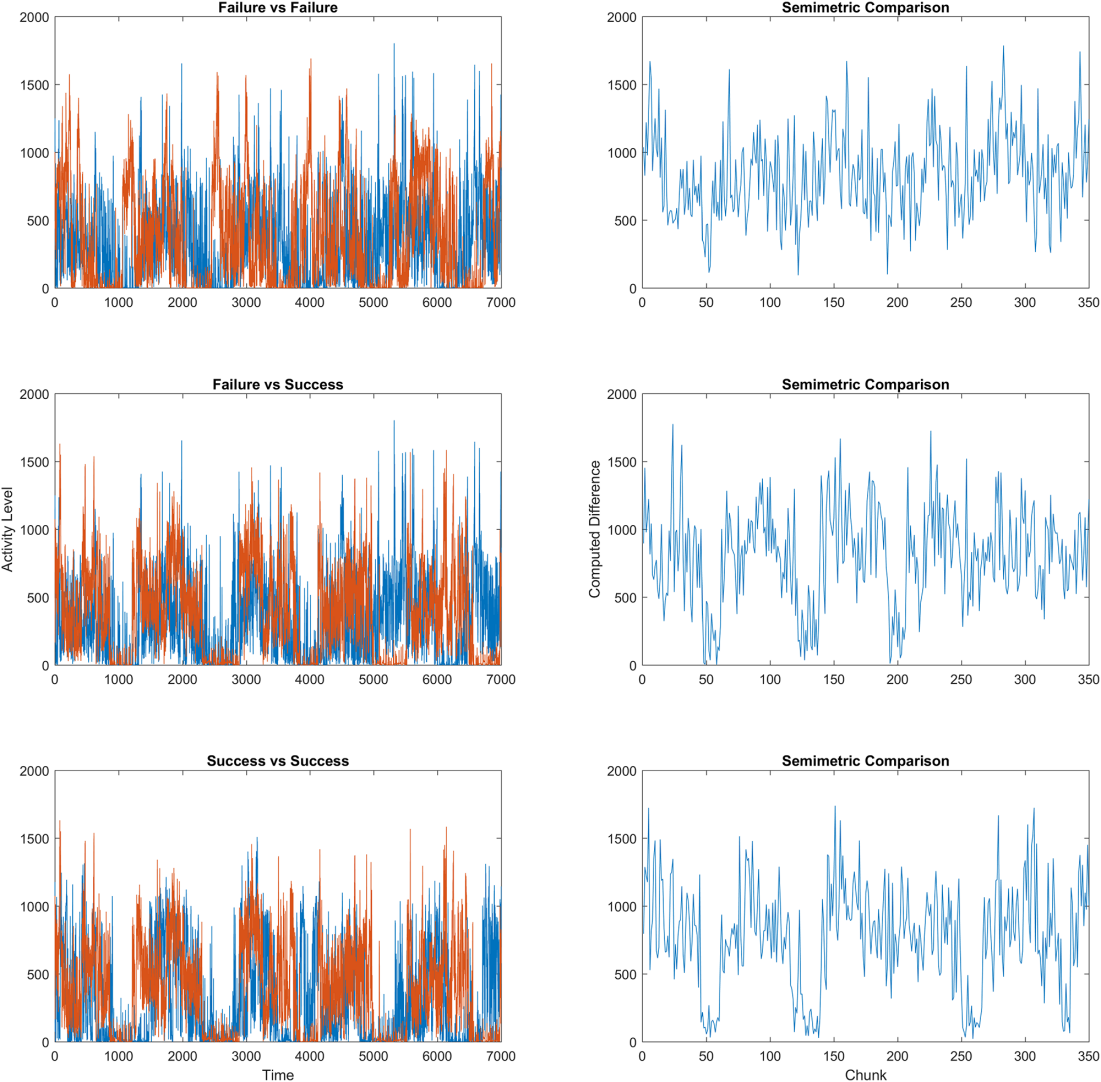
Signal comparisons.

Fig 3 shows an example comparison from each of the three groupings. As shown in the plots, the general movement profile recorded by the armband sensors varies more heavily within the failure group than in the success group. Intuitively, this indicates that those destined to fail behave in a variety of different ways while those who will lose weight and maintain the loss share a more unified pattern of behavior. The general topographical structures present in the signals represent these overall patterns of activity and are captured in the persistence diagrams; in this sense, persistent homology is ideally suited to uncovering the underlying differences. Because of the larger variation present across the movement profiles of the ‘failures’, the signal-to-signal comparisons yield consistently higher values of both the average distance and the corresponding standard deviation across all metrics (true and semi-) used above.

In utilizing our persistent homology algorithm, we are extracting a set of connected components from the time-series data [24]. In looking at this homology group, we are examining the underlying patterns of each individual’s short-term behavior. Intuitively, this shows that the types, frequencies, and amplitudes of movement vary between each group, not just in general trends but also in minute-to-minute fluctuations. Future studies of this phenomenon could lead to discoveries pertaining to physical movement and how it contributes to and informs future weight loss success.

While further analyses with more participants would greatly help strengthen and validate these results, the initial implications are twofold. First, the clear disparity between the two groups indicates that there is a measurable difference in the movement profiles of those that will lose weight and keep it off versus those that will not lose any or, after losing weight, regain a substantial amount. By measuring this contrast a patient could potentially be classified as a ‘success’ or a ‘failure’ before even beginning a diet, in turn leading to more effective and individually tailored interventions. This would help to greatly ease the economic costs associated with overweight and obesity, as well as their related diseases. It would also save both the clinician and the patient time spent pursuing a course of action likely to produce unsatisfactory results, instead allowing them the option to first pursue alternatives. Secondly, and more immediately evident, the results indicate that the windowed persistent homology method, coupled with the modified Hausdorff semimetric, is capable of detecting subtle, underlying differences between signals. This method could potentially be used in other clinical settings where a deeper analysis of a complex signal would result in improved care, as well as other signal processing applications. Relatively tolerant of noise and sampling frequency, our algorithm can be easily applied to short and long time-series alike, drawing out features from the signal useful in exposing subtle differences.

While the exact physical characteristics measured by the persistence diagram remain unclear and will be closely examined in future work, it can be said that the patterns representing more scattering in the persistence diagram represent higher levels of physical activity. In addition to further exploring the physical and physiological implications of an individual persistence diagram, there are a number of modifications to the implemented algorithm that we will investigate in future work. For instance, in our analysis, the inputs were blindly compared. In our future work, we intend to implement a set of alignment procedures in the pre-processing steps. Syncing time of day or sleep/wake cycles between two armband signals before the persistent homology algorithm is applied could help reduce any noise associated with comparing across states (e.g. one participants sleep data with another’s morning routine). Another route for our future investigations will be to implement a dynamic windowing method: by altering the length of each window based on the number of included extrema, we can improve the resolution of our algorithm in areas of high activity (e.g. exercise) without sacrificing accuracy in low-movement periods (e.g. sleep). A third future improvement to the analysis involves error-checking the edge cases. If a window boundary splits a monotonically increasing or decreasing section of the signal, a false maximum and minimum are formed on either side. By implementing a check on the next point outside any given window, we can reduce the number of artificial extrema, thus reducing inaccurate pairings and noise in the persistence diagrams.

## Conclusions

To predict *a priori* weight loss/maintenance success in overweight or obese individuals by applying information learned from one week of simple, noninvasive measures would heavily impact the healthcare industry. With such a high prevalence rate in the country, both the economic burden and the time spent treating overweight, obesity, and their related diseases could be drastically reduced. By facilitating more tailored and individualized treatment, which may include modifying an existing program, identifying alternative modalities, or focusing on other patient issues, countless hours could be saved for both the patient population and the clinicians. The results presented in this paper indicate, through the use of a novel computational method, a measurable contrast between the group of participants able to maintain weight loss and the group unable to do so. By using the windowed persistent homology method defined above and the modified Hausdorff semimetric, a physician could determine whether or not a specific intervention would be effective for a given patient. This project demonstrates the effectiveness of the novel signal processing method and the potential impact it can have on clinical decision making and patient care.

## Supporting information

S1 File Raw data. This file contains the raw data analyzed in the paper.
(CSV)
